# Development and validation of a face database for the recognition of facial expressions of basic emotions in the Brazilian population

**DOI:** 10.1186/s41155-024-00325-y

**Published:** 2024-12-02

**Authors:** Daiene de Morais, Madson Alan Maximiano-Barreto, Bruna Moretti Luchesi, Monalisa Muniz, Marcos Hortes Nisihara Chagas

**Affiliations:** 1 Department of Psychology, Federal University of São Carlos, São Carlos, SP Brazil; 2 Study and Research Group in Mental Health, Cognition and Aging, University of São Paulo, Ribeirão Preto, Brazil; 3 Department of Gerontology, Federal University of São Carlos, São Carlos, SP Brazil; 4 Três Lagoas Campus, Federal University of Mato Grosso Do Sul, Três Lagoas, MS Brazil; 5 Research Group On Emotional Intelligence, Federal University of São Carlos, São Carolos, SP Brazil; 6 Neurosciences and Behavioral Sciences Department, University of São Paulo, Ribeirão Preto, SP Brazil

**Keywords:** Facial expressions of emotions, Social cognition, Face database, Psychometric qualities

## Abstract

**Background:**

The recognition of facial expressions of emotions is an essential skill for social functioning, as it enables recognizing the possible intentions of others.

Main body.

Cultural context is an important aspect to consider in this skill, as it tends to modulate the recognition of facial expressions.

**Objective:**

To develop and validate a set of facial expressions of basic emotions of Brazilian individuals considering the population distribution of the country according to age group, sex, and race.

**Methods:**

A procedure with three phases was created to generate basic emotions and photograph facial expressions. A total of 459 Brazilians were then asked to identify the emotions of these facial expressions. Content validity was investigated based on the analysis of specialists and the recognition of emotions by Brazilian individuals.

**Results:**

The final database consists of 56 high-quality color images. A good level of agreement was found for the expressions perceived by the assessors (81.6%).

**Conclusion:**

The percentage of correct recognition of each emotion and the characteristics of the models are presented so that future studies can choose the most adequate images to meet their specific needs.

**Supplementary Information:**

The online version contains supplementary material available at 10.1186/s41155-024-00325-y.

## Introduction

Emotions play an important role in social interactions and can be characterized as a momentaneous phenomenon of physiological and psychological changes in response to situations of personal importance. One of the purposes of emotions is to facilitate social adaptation (Gendron et al., 2014; Miguel et al., 2020). Nonverbal communication (i.e., our intentions and emotions) is largely expressed through the face (Palermo & Rhodes, 2007). Thus, the capacity to recognize facial expressions of emotions is directly related to success in social interactions (Lacerda, 2010), contributing to the management of daily social situations that seem essential for the maintenance of wellbeing and are directly related to our adaptive process (Ekman & Cordaro, 2011).

Evidence has shown that basic emotions (i.e., happiness, sadness, fear, disgust, anger, and surprise) are expressed and recognized in different cultures (Ekman & Friesen, 1971; Izard, 1994). However, different cultural groups can have differences in the response pattern for the recognition of specific emotions (Jack et al., 2009; Matsumoto & Assar, 1992). For instance, individuals in Western cultures have greater activation of the amygdala (brain structure that plays an important role in the recognition of facial expressions) when recognizing faces expressing fear in members of their own cultural group (Chiao et al., 2008). One study involving Caucasian and Chinese individuals asked to categorize emotions of happiness and anger based on stimuli of the same race, opposite race, males and females in separate tasks found that the “happiness” stimulus on male faces of the same race had a higher number of correct responses (“hits”) compared to faces of the other race (Craig et al., 2017). A lower number of “hits” was found when men and women categorized faces of “happiness” of the same race but opposite sex. Thus, the influence of social group factors in the emotion recognition task may exert an influence on the recognition of facial expressions of emotions (RFEE) (Craig et al., 2017).

Besides race and sex, studies have sought to identify whether RFEE is also modulated by the age of the stimulus presented in the task. The “hit” rate is higher when categorizing emotions on young faces compared to faces of older people (Ebner, 2008; Richter et al., 2011). However, characteristics other than age, race, and sex can modulate how we recognize emotions. This complex skill also requires flexibility and sensitivity to the context (Spunt et al., 2017). Due to the influences of the cultural context on RFEE, it is important for studies to use stimuli created in accordance with this context.

A study conducted in a Brazilian city produced prototypes of the faces of individuals of the most common races (white, black, and brown) in the city in which the study was conducted rather than of the entire country (Mendes et al., 2009). Other studies used different races but only used the faces of infants (Donadon et al., 2019) of children (Negrão et al., 2021) and did not consider the Brazilian distribution according to these variables. Although studies have sought to create face databases to enable the assessment of the recognition of basic facial emotions in specific contexts, there is no database that has taken into consideration the population distribution of the country according to age, sex, and race in the creation and applicability of stimuli to be used in Brazil.

Performance on an emotion recognition task can be affected by different factors, such as the participant’s background and the characteristics of the stimuli presented. Cultural elements, such as race, sex, and age group, seem to be relevant aspects for the recognition of emotions through facial expressions. Considering the broad spectrum of these elements in Brazil, it is of extreme importance for tasks that assess RFEE to take these aspects into consideration. Therefore, the aim of the present study was to create and validate a set of facial expressions of basic emotions of Brazilian individuals considering the population distribution of the country according to age, sex, and race.

## Materials and methods

### Transparency and openness

This study’s design and its analysis were not preregistered. The present study was divided into the following steps: (1) creation of the procedure to generate basic emotions; (2) creation of a database of faces of different ages, sexes, and races; (3) investigation of the validity of the database based on the analysis of specialists and editing of the face database; (4) investigation of content validity of the face database with the participation of Brazilian individuals. Each step has different methodological characteristics, which are presented below.

1. Creation of procedure to generate basic emotions.

For the creation of the procedure to generate basic emotions in the participants, we performed a systematic review of the literature (Fabrício et al., 2022) to unite studies involving the creation of face databases for the assessment of the recognition of basic emotions, describing and comparing the methods used in the creation of these stimuli. This enabled establishing important patterns that studies used in the creation of face databases. Given the importance of using more than one stimulus to generate the most spontaneous emotion possible, we opted to induce the emotions using the following three steps: (1) Presentation of specific stimuli of the International Affective Picture System (IAPS) (Lang et al., 1997); (2) Presentation of model photographs of the Pictures of Facial Affect (POFA) database (Ekman & Friesen, 1976)); (3) Specific hypothetical situations (Yang et al., 2020).

For the selection of stimuli to be used in each phase, we invited 26 specialists/researchers in the fields of mental health and psychology. Most were women (84.7%) and age ranged from 22 to 39 years. Participants had to fill a form in the *Google Forms* platform. A pilot study was then conducted with six volunteers (three women and three men; average age of 28.3 years) selected by convenience to test the understanding of each phase. All participants understood the objective of the study and the steps to express basic emotions. A professional tripod was used to hold the camera at a standardized angle. The images were captured with a white background and white light for the standardization of illumination. The different phases are described below.

#### Phase 1: Presentation of specific stimuli from IAPS

Thirty-five images were selected based on basic emotions most frequently listed by men and women when using the specific stimuli of IAPS, which consists of a collection of 1196 photographs used for experimental investigations of emotion and attention in national and international studies found in the “Handbook of emotion elicitation and assessment” (Coan & Allen, 2007). The images are divided into categories (e.g., animals, family, nature, pollution, accidents) and experienced emotions (e.g., happiness, sadness, disgust, anger). We selected the categories that generated the emotions of interest in the present study.

The participants were asked to choose the emotion corresponding to each of the IAPS stimuli. An image was presented and the participants were to select which emotion (happiness, sadness, fear, disgust, anger, surprise, or neutral) the stimulus made them feel. Stimuli with a higher percentage of agreement among the participants were selected to compose the items of the procedure (analysis generated by the platform itself). We concluded this phase with a photo of the IAPS to generate each of the intended basic emotions.

#### Phase 2: Presentation of model photographs of the POFA database

We randomly selected a stimulus of each emotion from the POFA database of basic emotion (Ekman & Friesen, 1976), which is one of the pioneers and most widely used databases for the recognition of facial emotions. In this procedure, the model stimulus was presented, and the volunteers were instructed to express the same emotion presented in the image.

#### Phase 3: Specific hypothetical situations

Following the same procedure as in Phase 1, we used the hypothetical situations used by Yang et al. (2020) to “test” what scenarios were capable of provoking the intended emotion as much as possible. For instance, for “happiness,” the volunteers were to select scenarios that would be capable of provoking the emotion to the maximum: “You just won 500 million in the lottery!” or “You just got the job offer of your dreams!” or “Someone you like just declared their love to you!” or “You are meeting your best friend who you haven’t seen in a very long time!” or “You are having a wonderful time with your loving family!” or “You are celebrating your best friend’s wedding or birthday!”. The options for each of the basic emotions were presented, as described by Yang et al. (2020). We included situations with the highest percentage of agreement and that most provoked the specific emotions among the participants.

2. Creation of face database considering age, sex, and race.

In this step, individuals were selected for the creation of the face database. Volunteers with acting experience (amateurs and professionals) were selected by convenience from theater companies in the city. As it was not possible to find all ages and races at the theater companies, we opted to include non-actors from the community in the database to ensure that the characteristics of the volunteers were representative of the population distribution of Brazil.

The proportion of each group for age, sex, and race was based on the population distribution in the country (BRAZIL, 2010). Thus, approximately 51% of the faces needed to be female, 48% needed to be of individuals with self-declared white skin color, 43% needed to be of individuals with self-declared brown skin color, 8% needed to be of individuals with self-declared black skin color, 1% needed to be of individuals with self-declared Asian descent and 0.5% needed to be of self-declared indigenous individuals. In terms of age, 52% needed to be of individuals 18 to 39 years of age (young adults), 33% needed to be of individuals 40 to 59 years of age (middle-aged adults), and 15% needed to be of individuals 60 years of age or older (older adults). To meet the population distribution criteria in each group, the face database was created with 29 volunteers*.*

**Fig. 1:**
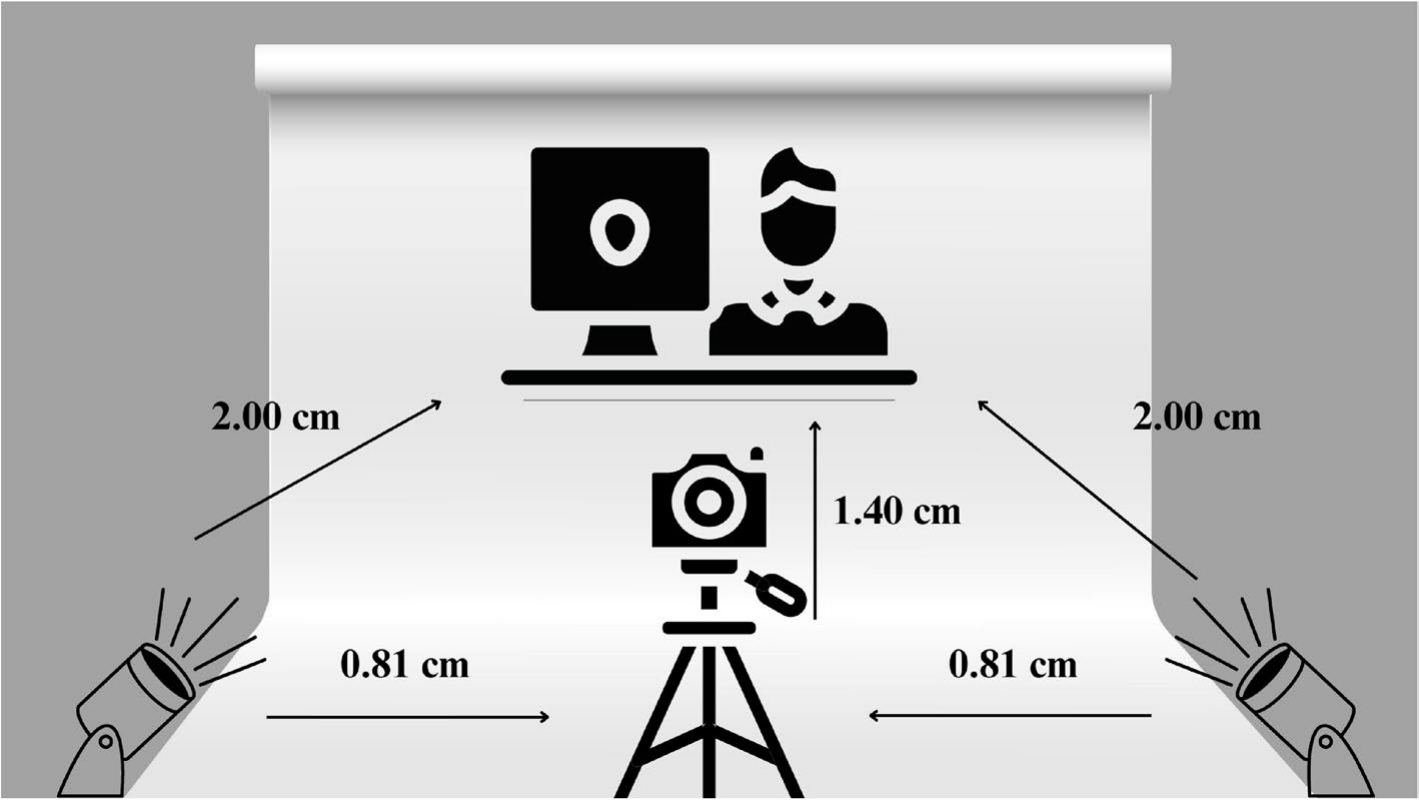
Scheme of equipment positioning in the stimulus construction stage

The reactions of the participants to the stimuli in the three phases were recorded with a Canon Sl2 camera. All models wore a black t-shirt and were positioned in front of a white background. We also instructed the volunteers not to use any type of distracting objects, such as earrings, makeup or piercings. We extracted individual static frames from the original video made to capture each emotion. A schematic of the scenario is presented in Fig. 1 below. We concluded this step with 704 images of faces, 28 of which were excluded due to inadequate quality.

3. Evidence of validity based on analysis of specialists and editing of database.

The aim of this step was to determine evidence of content validity for the faces recorded in the previous step. Five specialists in studies involving emotions and psychometrics (three with doctoral degrees, one with a master’s degree, and one doctoral student—three women and two men) were invited to participate as judges. The judges' experience was based on articles published in the area and/or research projects on the topic. Using a form created in Google Forms, the specialists were to determine the emotion that each model was expressing. A total of 676 images were presented one by one in random order. Besides marking the emotions that the model was expressing, the judges were also asked to categorize the difficulty in recognizing each emotion (easy, medium, or difficult).

Among the 676 faces, 286 were recognized with 100% agreement among the judges for the type of emotion that was being expressed by the volunteer (happiness: 89; sadness: 36; fear: 17; disgust: 57; anger: 39; surprise: 31; neutral: 17). Agreement was not 100% for all levels of difficulty in most cases. Thus, the criteria for each emotion in still frame to be included in the final database were faces where: (1) agreement was 100% or 80% for the easy category; (2) also, when this number did not reach a minimum of eight items (faces), we included faces recognized by the majority of judges as having a medium level of difficulty; and/or (3) we completed the items taking into consideration the variables of interest, which resulted in a final database of 56 faces—eight faces for each basic emotion and eight neutral faces. Faces categorized by judges as “difficult” were not included in order to avoid possible bias in the recognition of these faces by the sample in the subsequent validity step; reported a high level of difficulty in recognizing the emotions, these faces could also be confusing to the general population.

**Fig. 2:**
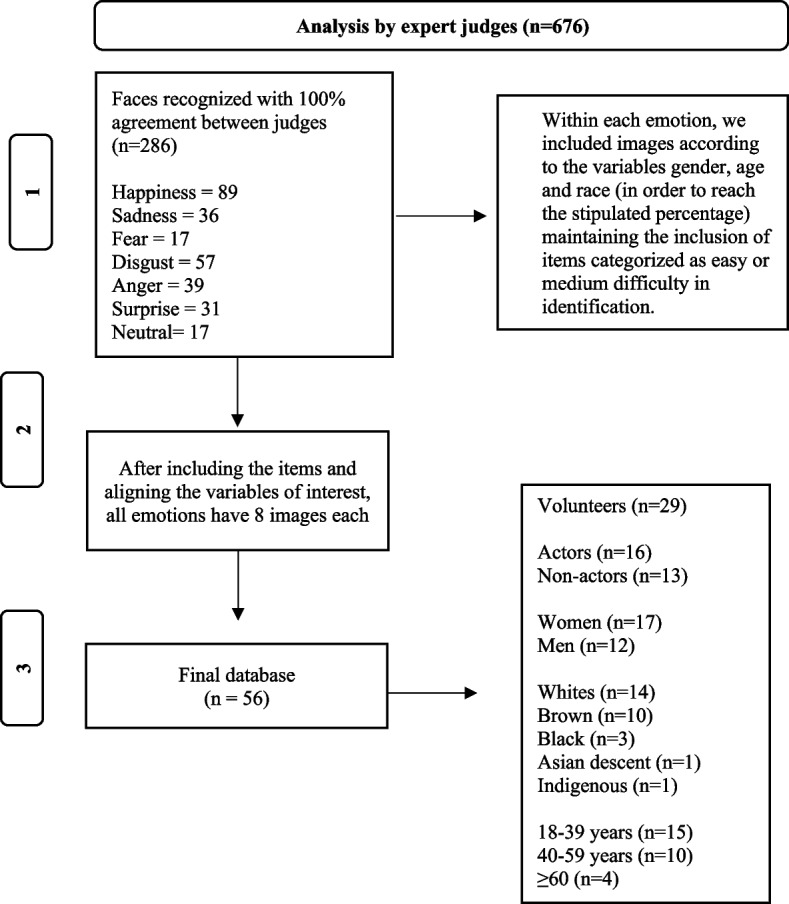
Image selection process and analysis by expert judges

Figure 2 below presents the image selection process and the composition of the database in accordance with the objectives.

**Fig. 3:**
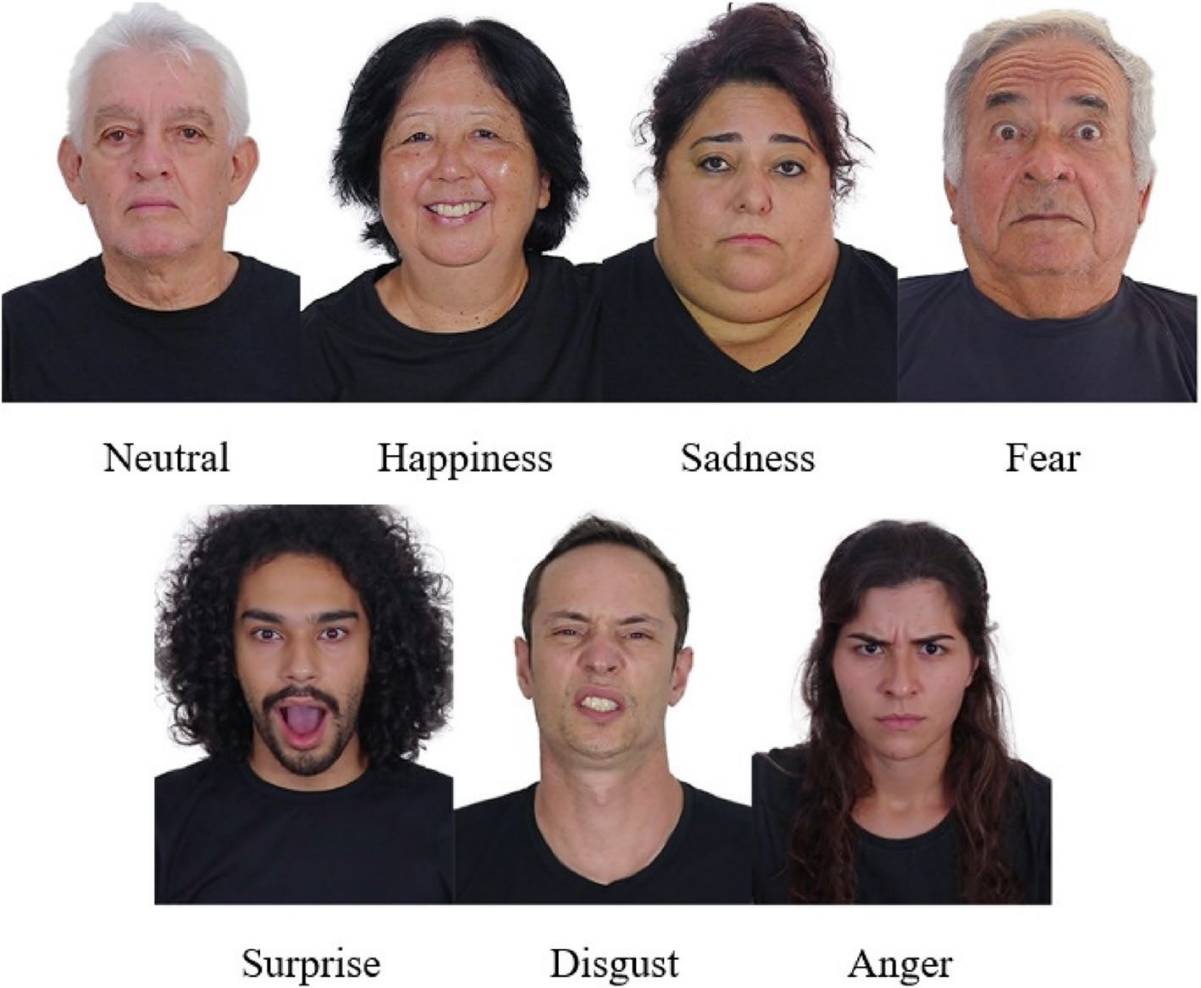
Examples of basic emotions and neutral face

All 56 images were edited by a professional company using the Adobe Photoshop software, employing standardized sizes (270 × 360 pixels), positions (rule of thirds—eyes aligned in the upper horizontal third, top of head near the upper margin and nose centralized horizontally) and lightning (temperature, color, contrast, shadows, whites, blacks, and vibration). Piercings and dental applications were removed from three volunteers. Figure 3 presents an example of the basic emotions constructed in some participants.

4. Evidence of content validity of face database in Brazilian individuals.

This step was conducted to determine whether a sample of the Brazilian population would be able to identify the expressions, offering further evidence of content validity.

For this step, the study was publicized in a public call on the university radio and social media. The volunteers were asked to visit a room in the psychology department of the university or a place of their choice. Upon approval of the study by the ethics committee to be run online, the experiment was made public in social media, and data collection was carried out 100% online (via *Google Forms*). Data collection took place between August 10th and September 28th, 2022. A total of 459 volunteers aged 18 years or older were selected by convenience.

The online form included the statement of informed consent and sociodemographic data for the characterization of the sample (age, biological sex, marital status, race, educational level, and number of residents in the home). We also used the Patient Health Questionnaire-2 (PHQ-2) to screen for symptoms of depression, which could affect the recognition of emotions (Bourke et al., 2010; Dalili et al., 2015). The PHQ-2 is widely used in Brazil (Chagas et al., 2011; De Lima Osório et al., 2009). The score ranges from zero to six points and a score of three is used as the cutoff point for major depression disorder.

The 56 images were presented to the volunteers, who were instructed to choose the emotion that each individual was expressing through multiple choice options. No time restriction was imposed. The entire process took approximately 15 min to complete.

All steps of this study received approval from the Human Research Ethics Committee of *Universidade Federal de São Carlos* (UFSCar) (process numbers: 30253420.0.0000.5504 for in-person modality and 61,477,722.2.0000.5504 for online modality). After clarifications regarding the procedures, all participants provided informed consent.

### Data analysis

Descriptive analyses (frequency, mean, and standard deviation [SD]) were performed to categorize the sample in a general manner and according to sociodemographic variables. The percentage of “hits” was calculated for each emotion. Pearson’s chi-square test was used for categorical data to describe the performance on each item according to age, sex, educational level, race, marital status, number of residents in the home, and PHQ-2 score.

The Kolmogorov–Smirnov test was used to determine the normality of the continuous variables (age, total performance on faces, and per emotion) (*p* ≤ 0.05). As the distribution of these data was non-normal, nonparametric statistical tests were used. The Kruskal–Wallis test was used to compare the performance on each emotion according to race, marital status, educational level, and number of residents in the home. We also used the Mann–Whitney *U* test for pairwise comparisons to further examine eventual detected differences. This test was also used to investigate differences in performance between two independent samples (individuals with and without depressive symptoms based on the PHQ-2; males and females) to provide evidence of validity based on the relationship with other variables.

Cohen’s *D* was used to calculate the effect size when differences were found. The coefficients were interpreted as follows: 0.2–0.3 = small effect size; 0.5–0.8 = medium effect size; > 0.8 = large effect size (Cohen, 1988).

The analysis of the precision of the set of facial expressions and each item of the database was performed using Cronbach’s alpha (α)^29^ and McDonald’s omega (ω) (McDonald, 2013), considering values ≥ 0.60 to be acceptable (Andrade et al., 2018) and ≥ 0.70 to be ideal (Campo-Arias & Oviedo, 2008; Viladrich et al., 2017). All analyses were performed with the aid of the Statistical Package for the Social Sciences (SPSS, version 23), Jeffreys’s Amazing Statistics Program (JASP, version 16.4), and Microsoft Excel. The significance level for all analyses was set at 5% (*p* ≤ 0.05).

## Results

### Characterization of face database

**Fig. 4:**
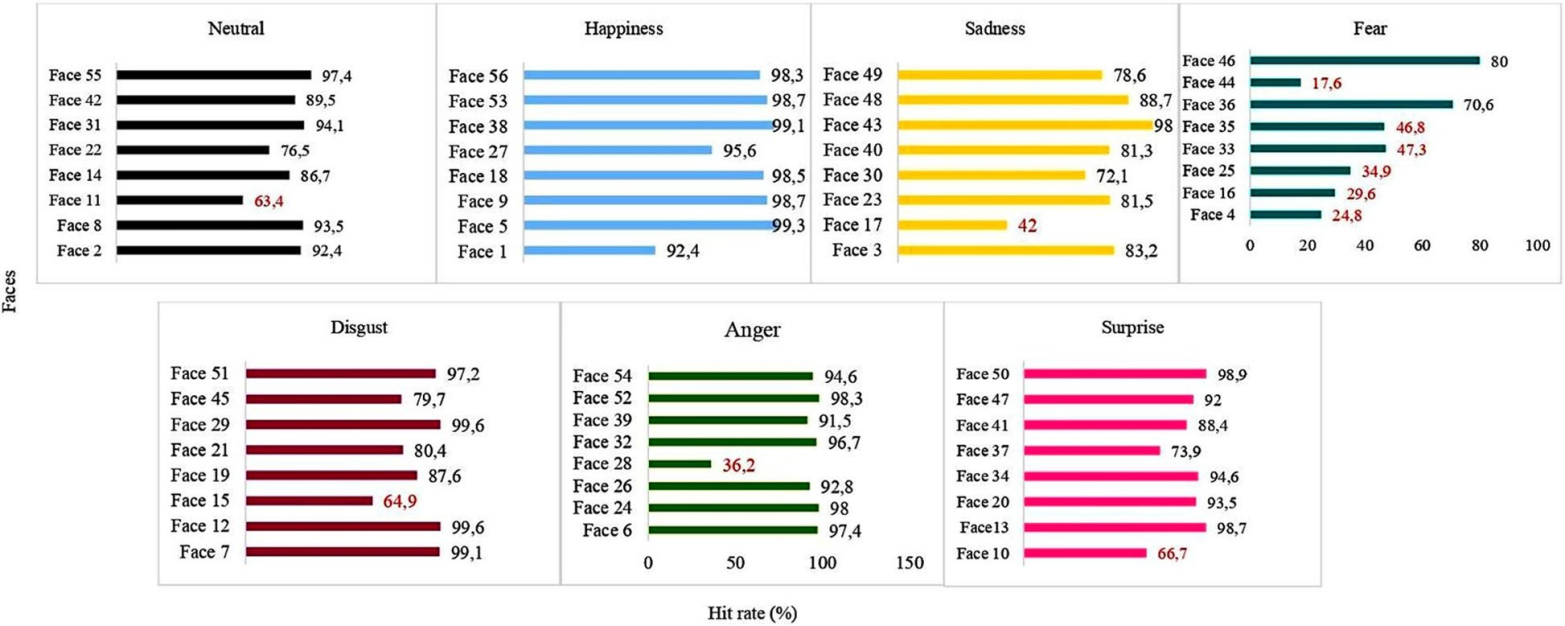
The final face database constructed

The ensure the compatibility of the characteristics and the population distribution in Brazil and taking into consideration the analyses of the judges and objectives of the present study, it was not possible to have a single volunteer express all basic emotions despite having all physical characteristics present in the overall database. The final face database was composed of 56 stimuli expressed by 29 volunteers, 16 of whom were actors (amateurs and professionals). Figure 4 shows the final face database constructed. The number of volunteers was based on the population distribution of the most recent Brazilian census for sex, age, and race^23 ^Brasil (2010), and the characteristics of each face that makes up the database are presented in the supplementary material.

### Characterization of sample in content validity step

Table 1 displays the sociodemographic characteristics of the 459 volunteers who participated in the content validity step. Women accounted for 74.9% of the sample. Average age was 33.08 years (SD: 11.13), with a minimum age of 18 years and maximum of 70 years. Most participants were single (53.5%) and had self-declared white skin color (73%). A total of 39.6% of the volunteers had a postgraduate degree and 30.9% reported living with another person in the home. The presence of depressive symptoms (score ≥ 3 on PHQ-2) was identified in 38.6% of the participants.

**Table 1:** Characterization of the sample from the validity evidence stage of the constructed face database

**Sex**	***N***	**%**
Female	344	74.9
Male	115	25.1
**Age**	***N***	**%**
18–39 years	238	51.9
40–59 years	200	43.5
60 years or more	21	4.6
**Marital status**	***N***	**%**
Single	246	53.5
Married	188	41
Divorced	22	4.8
Widower	3	0.7
**Race**	***N***	**%**
White	335	73
Brown	86	18.7
Black	25	5.4
Asian descent	9	2.0
Indigenous	4	0.9
**Education**	***N***	**%**
Postgraduate	182	39.6
Complete higher education	133	29.0
Secondary education (up to 11 years of schooling)	135	29.4
Elementary education (up to 8 years of schooling)	9	2.0
**Housing**	***N***	**%**
Alone	61	13.3
One more person	142	30.9
Two more people	130	28.3
Three people or more	126	27.5
**PHQ-2**	***N***	**%**
No depressive symptoms	282	61.4
With depressive symptoms	177	38.6
**Total**	**459**	**100%**

### Overall performance on recognition of emotions

We first calculated the average number of “hits” for each emotion and for the entire database and then calculated the percentage of correct identification (based on the number of times that the facial expressions were correctly identified by the participants), which reflects the level of identifiability of the facial expressions. Table S2 (Supplementary Material) displays the average performance on the 56 faces and according to each emotion (eight items of each) of the overall sample (*n* = 459) in recognizing the basic emotions expressed in the image database.

The highest “hit” rate was found for “happiness” (mean; 7.8; SD: 0.53) and the lowest was found for “fear” (mean: 3.52; SD: 1.95). A total of 43.6% of the faces that should have been attributed to “fear” were attributed to “surprise.” The only emotions with a nil “hit” rate were “fear” and “neutral.” We describe the performance for each emotion in each group according to the variables analyses in the following section.

The supplementary material details the performance in recognizing each emotion that makes up the face database according to each group for the variables age, sex, race, presence of depressive symptoms, education, marital status, and number of residents in the house (*n* = 459).

### Performance according to each item of the image database

**Fig. 5:**
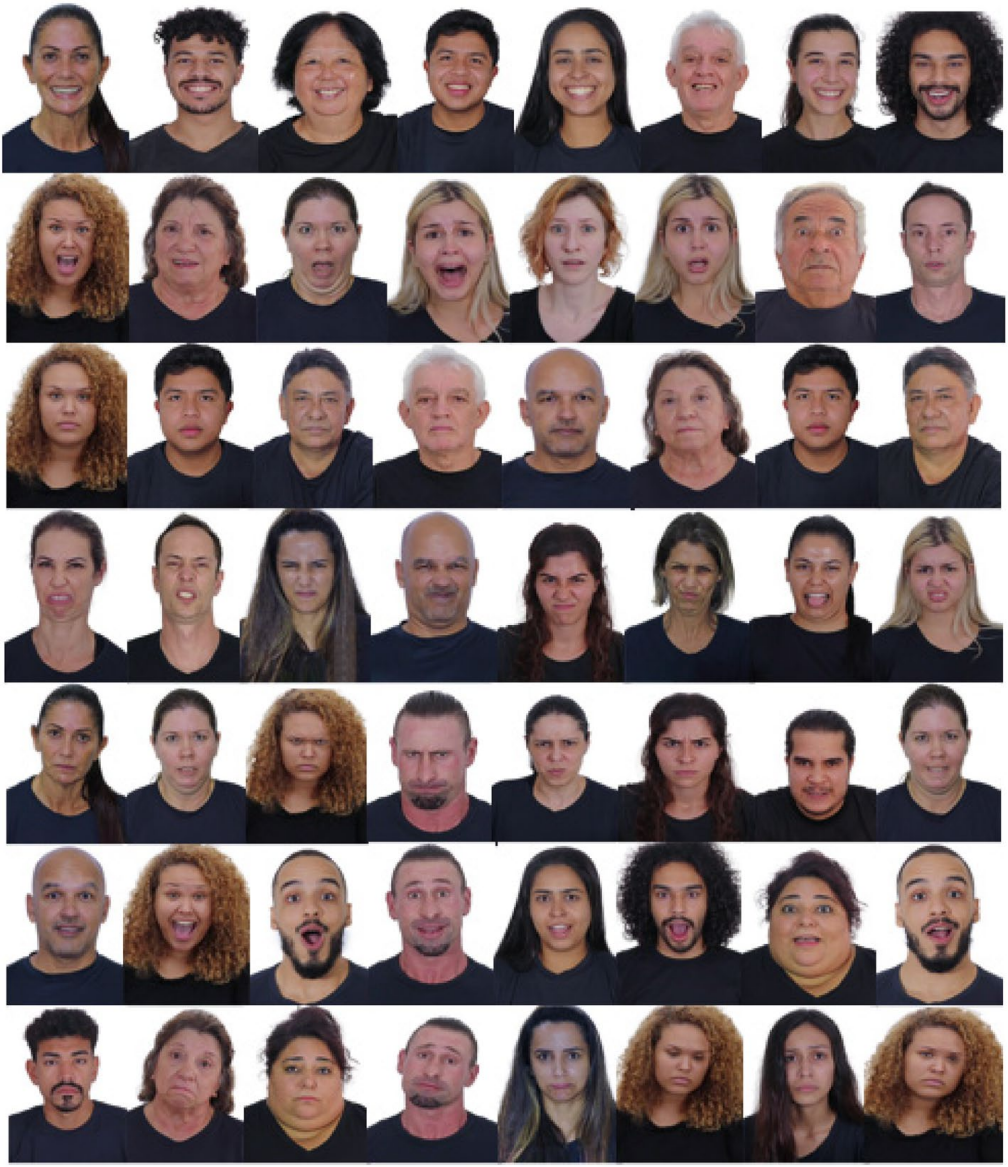
Performance of the 459 participants in the recognition of the eight faces by emotion presented (i.e., neutral, happiness, sadness, fear, disgust, anger, and surprise)

Figure 5 displays the performance of the overall sample (*n* = 459) on each emotion for all faces individually. Percentages in red type refer to faces for which the “hit” rate was < 70%.

The emotions “sadness,” “disgust,” “surprise,” and “neutral” had one face for each emotion with a performance lower than 70% (faces 10, 11, 15, and 17, respectively). For “fear,” performance was unsatisfactory only for faces 36 and 46. In the Supplementary Material, it is possible to visualize all these faces. The results of the comparisons based on the characterization variables are presented below.

- Age: Significant differences with a large effect size were found in the performance between young adults and middle-aged adults for the emotion “happiness”; between young adults and older adults and between the middle-aged adults and older adults for “surprise”; between young adults and middle-aged adults and between young adults and older adults for “neutral”. With regards to the performance for the entire database, differences were found between young adults and older adults and between the middle-aged adults and older adults.
- Sex: Significant differences with a small effect size were found for “sadness” and “fear.”
- Race: Significant differences with a small effect size were found between white and brown individuals and between white and black individuals for “disgust.” The effect size was large in the comparison between blacks and individuals of Asian descent. Moreover, significant differences were found between white and brown individuals and between brown and black individuals (small effect size) for the overall database.
- Schooling: Significant differences with a large effect size were found in groups with different educational levels for “sadness” and the overall database. With regards to “sadness,” differences were found between groups with a primary school and high school education and between groups with a primary school and postgraduate education. For the performance on the overall database, differences were found between groups with a primary school and complete higher education, between groups with a primary school and postgraduate education, and between groups with a higher education and postgraduate education.

No significant differences between groups were found for marital status, number of residents in the home, or the presence of depressive symptoms. The data described above are detailed in the supplementary material.

Cronbach’s alpha for the set of facial expressions was 0.70 and McDonald’s omega was 0.68. The analysis of the accuracy of each item is described in Table 5 of the Supplementary Material. Both Cronbach’s alpha and McDonald’s omega were satisfactory for all items.

## Discussion

In the present study, we constructed and validated a multiracial database of stimuli of facial expressions of basic emotions with high-quality color photographs of 29 men and women of different ages (young adults, middle-aged adults, and older adults). Each item was assessed by five specialists and 459 Brazilian adults. The results revealed a good level of agreement of the expressions perceived by the assessors and the expression of each face in the items that compose the database (mean: 81.6%), which is compatible with findings described in previous face database studies (Dalrymple et al., 2013; Ebner et al., 2010; Yang et al., 2020). A higher “hit” rate was found for the emotion “happiness” (mean: 97.63%). The same result was reported in other studies that created face databases (Ebner et al., 2010; Yang et al., 2020).

Behavioral studies found that faces expressing emotions of happiness were identified with greater precision and more quickly than all other emotions (Svard et al., 2012; Tottenham et al., 2009). This ease in recognizing happy faces may be largely due to the clear differentiation of the basic emotion in relation to other basic emotions due to its positive valence (represented by the smile) (Rodger et al., 2015).

In contrast, the poorest performance was found in the identification of “fear” (43.91%), which was often confused with “surprise.” Similar results were reported in previous database construction studies (Dalrymple et al., 2013; Yang et al., 2020). These emotions share the common characteristics of the eyes being more open, which may contribute to the confusion, consequently requiring a greater degree of cognitive processing and interpretation to differentiate the two emotions precisely (Skuse, 2003).

Considering the inclusion criterion for items often adopted in database construction studies (≥ 70% agreement) (Dalrymple et al., 2013; Ebner et al., 2010; Tottenham et al., 2009; Yang et al., 2020), an ideal level of agreement was not reached for 11 faces in the present study. Although our results support the validity of the images included in the database, we suggest the replication of this study in samples with a more representative number of individuals in the age and race groups. The performance in recognizing each face and the characteristics of each model is presented so that future studies may choose the most adequate images to meet their specific needs.

By selecting specialists in the study of emotions to be judges, our objective was to include in the subsequent phase only faces recognized with 100% agreement for the type of emotion expressed by the model, following the same procedure as that reported in a previous database construction study (Negrão et al., 2021). Despite being a simple analysis method, agreement among judges with regards to the type of response is widely used in studies (Matos, 2014). For reference values, 75% is considered the minimum level of acceptable agreement and agreement above 90% is considered high (Stemler, 2019). Thus, the criterion to include only faces with 100% recognition agreement among the judges strengthens the content validity of the present face database.

The set of stimuli was created due to the need to develop a database that considered the population distribution in Brazil for variables that can exert an influence on the recognition of emotions, especially age, sex, and race (Craig et al., 2017; Ebner, 2008). It is important to consider the influence of the context in which we live on the way that we perceive emotions (Barret et al., 2011). Therefore, we presented the detailed characteristics of each face in the database to facilitate future experiments.

This study has limitations that should be considered. The objective of the creation of a face database was to include models who expressed the basic emotions and had characteristics of age, sex, and race compatible with the population distribution of Brazil. Although the number of participants who participated in the construction phase is not representative, we were able to achieve the age, sex, and race variables according to the distribution in our country, which was our main objective. Furthermore, it would have been useful if we had all these variables represented in all emotions (e.g., have faces of men and women in all age groups and races). However, not all models expressed emotions that achieved 100% agreement among the judges. Thus, although the final database had the expected variability, this did not occur within each emotion. It would also have been useful to have a database with a greater variability of older people and individuals of self-declared Asian descent and indigenous individuals. Secondly, when categorizing the images into easy, medium, and difficult difficulty, we end up finding a subjectivity of evaluation at this point among the judges, since this classification can vary and be very broad among them. Furthermore, although the validity step was performed in the online modality in order to have a greater number of participants, it was not possible to encompass all variables in a substantial manner. Thus, it is important to apply the face database in studies with a greater number of older people and self-declared black individuals, individuals of Asian descent, and indigenous individuals.

## Conclusion

The present study developed and presented evidence of the validity of a face database with 56 high-quality color images for which a good level of agreement was found for the expressions perceived by the selected judges and the general population. The “hit” rates for each face and the characteristics of the models are also presented so that future studies can choose the most adequate images to meet their specific needs.

We hope that future studies will use our database to assess the effect of these and other variables on the recognition of emotions in the Brazilian context, specifically addressing associations between the race of the model and race of the participant who recognizes the facial expressions. We also suggest that future studies consider the creation of dynamic stimuli.

## Supplementary Information

Additional file 1: Table S1 Characteristics of the final face database models. Table S2 Average performance on the 56 faces according to each emotion (eight items of each) of the overall sample (*n* = 459) in recognizing the basic emotions expressed in the image database. Table S3 Performance for each emotion in each group according to the variables age, sex, race and presence of depressive symptoms (*n* = 459). Table S4 Performance for each emotion in each group according to the variables education, marital status and housing (*n* = 459). Table S5 Analysis of the accuracy of each item in the set of facial expressions.

## Data Availability

The data analyses used for the present study can be requested from the corresponding author. The main results are described in this article and in the supplementary material.

## Abbreviations

RFEE: Recognition of facial expressions of emotions
IAPS: International Affective Picture System
POFA: Pictures of Facial Affect
PHQ-2: Patient Health Questionnaire-2
SD: Standard deviation
SPSS: Statistical Package for the Social Sciences
JASP: Jeffreys’s Amazing Statistics Program
