# Supplementary material for: Development and validation of a face database for the recognition of facial expressions of basic emotions in the Brazilian population
Source: Psicol Reflex Crit. 2024 Dec 2;37:47. doi: 10.1186/s41155-024-00325-y (PMC11609135; doi:10.1186/s41155-024-00325-y)
Supplement: Supplementary file 1 — Additional file 1: Table S1 Characteristics of the final face database models. Table S2 Average performance on the 56 faces according to each emotion (eight items of each) of the overall sample (n = 459) in recognizing the basic emotions expressed in the image database. Table S3 Performance for each emotion in each group according to the variables age, sex, race and presence of depressive symptoms (n = 459). Table S4 Performance for each emotion in each group according to the variables education, marital status and housing (n = 459). Table S5 Analysis of the accuracy of each item in the set of facial expressions. [file 41155_2024_325_MOESM1_ESM.docx]

**Supplementary Material**

**Table 1**

*Characteristics of the final face database models*

| **Faces-Emotion** | **Model** | **Acting experience** | **Age** | | | **Sex** | | **Race** | | | | |
| --- | --- | --- | --- | --- | --- | --- | --- | --- | --- | --- | --- | --- |
|  |  |  | **18-39**  **years** | **40-59**  **years** | **60 years or more** | **F** | **M** | **White** | **Brown** | **Black** | **Asian descent** | **Indigenous** |
| **1 - Happiness** | 1 |  |  | x |  | x |  | x |  |  |  |  |
| **2 - Neutral** | 2 |  |  | x |  |  | x |  |  | x |  |  |
| **3 - Sadness** | 3 | x | x |  |  | x |  |  | x |  |  |  |
| **4 - Fear** | 4 | x |  | x |  |  | x | x |  |  |  |  |
| **5 - Happiness** | 5 |  | x |  |  | x |  |  | x |  |  |  |
| **6 - Anger** | 6 | x |  | x |  |  | x | x |  |  |  |  |
| **7 - Disgust** | 7 | x | x |  |  | x |  | x |  |  |  |  |
| **8 - Neutral** | 8 |  | x |  |  |  | x |  |  |  |  | x |
| **9 - Happiness** | 9 | x | x |  |  |  | x |  |  | x |  |  |
| **10 - Surprise** | 3 | x | x |  |  | x |  |  | x |  |  |  |
| **11 - Neutral** | 10 | x |  |  | x |  | x | x |  |  |  |  |
| **12 - Disgust** | 11 | x |  | x |  | x |  | x |  |  |  |  |
| **13 - Surprise** | 12 |  | x |  |  |  | x |  | x |  |  |  |
| **14 - Neutral** | 13 |  |  | x |  |  | x |  | x |  |  |  |
| **15 - Disgust** | 14 | x | x |  |  | x |  | x |  |  |  |  |
| **16 - Fear** | 15 |  |  | x |  | x |  | x |  |  |  |  |
| **17 - Sadness** | 16 | x | x |  |  |  | x |  |  | x |  |  |
| **18 - Happiness** | 17 | x | x |  |  | x |  | x |  |  |  |  |
| **19 - Disgust** | 2 |  |  | x |  |  | x |  |  | x |  |  |
| **20 - Surprise** | 5 |  | x |  |  | x |  |  | x |  |  |  |
| **21 - Disgust** | 4 | x |  | x |  |  | x | x |  |  |  |  |
| **22 - Neutral** | 18 |  |  |  | x | x |  | x |  |  |  |  |
| **23 - Sadness** | 19 |  | x |  |  | x |  |  | x |  |  |  |
| **24 - Anger** | 20 | x | x |  |  |  | x |  | x |  |  |  |
| **25 - Fear** | 7 | x | x |  |  | x |  | x |  |  |  |  |
| **26 - Anger** | 21 | x |  | x |  | x |  | x |  |  |  |  |
| **27 - Happiness** | 8 |  | x |  |  |  | x |  |  |  |  | x |
| **28 - Anger** | 1 | x |  | x |  | x |  | x |  |  |  |  |
| **29 - Disgust** | 22 | x | x |  |  | x |  |  | x |  |  |  |
| **30 - Sadness** | 6 | x |  | x |  |  | x | x |  |  |  |  |
| **31 - Neutral** | 13 |  |  | x |  |  | x |  | x |  |  |  |
| **32 - Anger** | 14 | x | x |  |  | x |  | x |  |  |  |  |
| **33 - Fear** | 23 | x | x |  |  | x |  | x |  |  |  |  |
| **34 - Surprise** | 12 |  | x |  |  |  | x |  | x |  |  |  |
| **35 - Fear** | 24 |  |  |  | x |  | x |  | x |  |  |  |
| **36 - Fear** | 3 | x | x |  |  | x |  |  | x |  |  |  |
| **37 - Surprise** | 6 | x |  | x |  |  | x | x |  |  |  |  |
| **38 - Happiness** | 25 |  |  |  | x | x |  |  |  |  | x |  |
| **39 - Anger** | 15 |  |  | x |  | x |  | x |  |  |  |  |
| **40 - Sadness** | 26 | x | x |  |  | x |  |  | x |  |  |  |
| **41 - Surprise** | 27 |  |  | x |  | x |  | x |  |  |  |  |
| **42 - Neutral** | 8 |  | x |  |  |  | x |  |  |  |  | x |
| **43 - Sadness** | 3 | x | x |  |  | x |  |  | x |  |  |  |
| **44 - Fear** | 18 |  |  |  | x | x |  | x |  |  |  |  |
| **45 - Disgust** | 19 |  | x |  |  | x |  |  | x |  |  |  |
| **46 - Fear** | 7 | x | x |  |  | x |  | x |  |  |  |  |
| **47 - Surprise** | 2 |  |  | x |  |  | x |  |  | x |  |  |
| **48 - Sadness** | 18 |  |  |  | x | x |  | x |  |  |  |  |
| **49 - Sadness** | 27 |  |  | x |  | x |  | x |  |  |  |  |
| **50 - Surprise** | 28 | x | x |  |  |  | x |  |  | x |  |  |
| **51 - Disgust** | 29 |  |  | x |  | x |  | x |  |  |  |  |
| **52 - Anger** | 3 | x | x |  |  | x |  |  | x |  |  |  |
| **53 - Happiness** | 28 | x | x |  |  |  | x |  |  | x |  |  |
| **54 - Anger** | 15 |  |  | x |  | x |  | x |  |  |  |  |
| **55 - Neutral** | 3 | x | x |  |  | x |  |  | x |  |  |  |
| **56 - Happiness** | 10 | x |  |  | x |  | x | x |  |  |  |  |

**Table 2.**

*Average performance on the 56 faces according to each emotion (eight items of each) of the overall sample (n = 459) in recognizing the basic emotions expressed in the image database.*

| **Emotions** | **M (*SD*)** | Minimum | Maximum | | **% (*SD*)** |
| --- | --- | --- | --- | --- | --- |
| **Happiness** | 7.8 (0.53) | 3 | 8 | 97.63 (6.57) | |
| **Sadness** | 6.25 (1.48) | 1 | 8 | 78.29 (18.56) | |
| **Fear** | 3.52 (1.95) | 0 | 8 | 43.91 (24.36) | |
| **Disgust** | 7.08 (1.01) | 4 | 8 | 88.63 (13.37) | |
| **Anger** | 7.05 (0.87) | 2 | 8 | 88.43 (10.88) | |
| **Surprise** | 7.05 (1.15) | 1 | 8 | 88.46 (14.34) | |
| **Neutral** | 6.93 (1.35) | 0 | 8 | 86.77 (16.93) | |
| **Total** | 45.7 (4.31) | 22 | 55 | 81.6 (7.69) | |

**M:** Mean; **SD:** Standard Deviation

**Table 3**

*Performance for each emotion in each group according to the variables age, sex, race and presence of depressive symptoms (n=459).*

| **Variables** | **Emotions (M; *SD*)** | | | | | | | |
| --- | --- | --- | --- | --- | --- | --- | --- | --- |
|  | **Happiness** | **Sadness** | **Fear** | **Disgust** | **Anger** | **Surprise** | **Neutral** | **Total** |
| **Age**  18-39 years (n=238)  40-59 years (n=200)  60+ (n=21) | 7.87 (0.03)^a^  7.73 (0.05)^a^  7.81 (0.11) | 6.34 (0.09)  6.24 (0.11)  5.38 (0.42) | 3.61 (0.12)  3.44 (0.15)  3.14 (0.50) | 7.07 (0.07)  7.10 (0.08)  7.05 (0.18) | 7.08 (0.05)  7.08 (0.06)  6.57 (0.33) | 7.11 (0.07)^b^  7.09 (0.08)^c^  6.33 (0.37)^b,c^ | 7.11 (0.08)^a,b^  6.80 (0.10)^a^  6.29 (0.38)^b^ | 46.20 (0.24)^b^  45.46 (0.34)^c^  42.57 (1.14)^b,c^ |
| ***H*** | 7.93 | 5.43 | 1.96 | 0.54 | 2.22 | 6.55 | 8.33 | 12.03 |
| ***p*** | 0.02* | 0.07 | 0.38 | 0.76 | 0.33 | 0.04* | 0.02* | <0.01* |
| ***Effect size*** | 3.39 | - | - | - | - | 2.92^b^/2.83^c^ | 3.42^a^/2.98^b^ | 4.40^b^/3.43^c^ |
| **Sex**  Male (n=115)  Female (n=334) | 7.79 (0.49)  7.81 (0.55) | 5.84 (1.53)  6.39 (1.44) | 2.85 (1.71  3.74 (1.97) | 7.03 (1.06)  7.10 (1.07) | 7.00 (0.96)  7.07 (0.84) | 6.92 (1.20)  7.11 (1.12) | 7.06 (1.27)  6.89 (1.38) | 44.50 (4.18)  46.12 (4.28) |
| ***U*** | 19130 | 15486.50 | 14379.50 | 18838 | 19263.50 | 18081.50 | 18276 | 15077 |
| ***p*** | 0.69 | <0.01* | <0.01* | 0.89 | 0.75 | 0.10 | 0.16 | 0.05 |
| ***Effect size*** | - | 0.370 | 0.482 | - | - | - | - | - |
| **Race**  White (n=333)  Brown (n=88)  Black (n=25)  Asian descent (n=9)  Indigenous (n=4) | 7.80 (0.51)  7.78 (0.68)  7.92 (0.28)  8.00 (0)  8.00 (0) | 6.28 (1.55)  6.36 (1.15)  5.76 (1.45)  5.67 (1.58)  6.25 (1.50) | 3.59 (1.88)  3.28 (2.08)  2.84 (2.01)  4.67 (2.45)  3.75 (2.06) | 7.17 (1.05)^a,c^  6.80 (1.09)^a^  6.68 (1.14)^c,e^  7.56 (0.53)^e,g^  6.75 (0.50)^g^ | 7.04 (0.91)  7.08 (0.78)  7.12 (0.67)  7.11 (0.60)  7.25 (0.50) | 7.07 (1.17)  7.14 (1.00)  6.64 (1.32)  7.22 (0.83)  7.00 (1.41) | 6.95 (1.29)  6.78 (1.60)  6.92 (1.41)  7.67 (0.50)  7.50 (0.58) | 45.91 (4.36)^a^  45.22 (4.14)^a,f^  43.88 (3.97)^f^  47.89 (3.69)  46.50 (4.04) |
| ***H*** | 3.52 | 5.76 | 7.11 | 17.44 | 0.13 | 3.85 | 3.78 | 11.24 |
| ***p*** | 0.48 | 0.22 | 0.13 | <0.01* | 1.00 | 0.43 | 0.44 | 0.02* |
| ***Effect size*** | - | - | - | 0.346^a^/0.447^c^  0.803^e^/1.572^g^ | - | - | - | 0.162ª/0.330^f^ |
| **Presence of depressive symptoms**  Yes (n=177)  No (n=282) | 7.79 (0.51)  7.82 (0.55) | 6.37 (1.46)  6.18 (1.49) | 3.53 (1.89)  3.51 (1.98) | 7.02 (1.12)  7.12 (1.03) | 7.07 (0.83)  7.04 (0.89) | 7.12 (1.15)  7.03 (1.14) | 6.89 (1.37)  6.96 (1.34) | 45.78 (4.46  45.67 (4.22) |
| ***U*** | 23906 | 23086 | 24844.50 | 23904 | 24750 | 23157.50 | 23829.50 | 24283.50 |
| ***p*** | 0.56 | 0.33 | 0.38 | 0.36 | 0.79 | 0.28 | 0.20 | 0.90 |

**M:** Mean; **SD:** Standard deviation; **H:** Kruskall-Wallis test; **U:** Mann-Whitney test; **p:** Probability of significance; *****Statistically significant difference; **a:** Difference found between groups 1 and 2; **b:** Difference found between groups 2 and 3; **c:** Difference found between groups 1 and 3; **d:** Difference found between groups 1 and 4; **e:** Difference found between groups 3 and 4; **f:** Difference found between group 2 and 4, **g:** Difference found between group 4 and 5. The effect size was calculated using *Cohen's D* for parametric data. For nonparametric data, the Z value generated by the *Mann Whitney* or *Kruskall Whallis* test was divided by the value of the sample analyzed.

**Table 4**

*Performance for each emotion in each group according to the variables education, marital status and housing (n=459).*

| **Variables** | **Emotions (M; *SD*)** | | | | | | | |
| --- | --- | --- | --- | --- | --- | --- | --- | --- |
|  | **Happiness** | **Sadness** | **Fear** | **Disgust** | **Anger** | **Surprise** | **Neutral** | **Total** |
| **Education**  Elementary education - up to 8 years (n=9)  Secondary education - up to 11 years (n=135)  Completed higher education (n=133)  Postgraduate (n=182) | 7.56 (0.73)  7.84 (0.44)  7.77 (0.53)  7.82 (0.58) | 4.67 (1.66)^a,d^  6.24 (1.58)^a^  6.26 (1.39)  6.34 (1.41)^d^ | 2.67 (1.80)  3.41 (1.85)  3.36 (1.92)  3.75 (2.03) | 6.67 (1.23)  7.07 (1.05)  7.01 (1.09)  7.16 (1.05) | 6.67 (1.87)  7.04 (0.85)  7.09 (0.81)  7.06 (0.86) | 6.11 (1.62)  7.04 (1.11)  7.00 (1.27)  7.18 (1.03) | 6.33 (1.66)  6.82 (1.44)  6.99 (1.31)  7.02 (1.29) | 40.67 (5.87)^c,d^  45.47 (4.26)  45.46 (3.97)^c,e^  46.33 (4.33)^d,e^ |
| ***H*** | 4.66 | 9.30 | 6.24 | 3.73 | 0.46 | 6.40 | 3.24 | 15.32 |
| ***p*** | 0.20 | 0.03* | 0.10 | 0.29 | 0.93 | 0.09 | 0.36 | <0.01* |
| ***Effect Size*** | - | 0.969^a^/1.084^d^ | - | - | - | - | - | 0.956^c^/1.097^d^  0.209^e^ |
| **Marital status**  Single (n=246)  Married (n=188)  Divorced (n=22)  Widower (n=3) | 7.82 (0.54)  7.80 (0.52)  7.73 (0.55)  7.33 (1.16) | 6.33 (1.39)^d^  6.18 (1.56)^f^  6.55 (1.14)^e^  3.00 (1.00)^d,f,e^ | 3.63 (1.88)  3.42 (1.99)  3.41 (2.11)  1.00 (1.00) | 7.07 (1.11)  7.09 (1.01)  7.14 (1.08)  7.00 (1.00) | 7.11 (0.77)^c,d^  7.06 (0.87)^f^  6.64 (1.09)^c^  5.00 (2.65)^d,f^ | 7.10 (1.10)  7.04 (1.20)  6.91 (1.27)  6.67 (1.16) | 7.03 (1.25)  6.86 (1.48)  6.68 (1.25)  6.00 (1.73) | 46.09 (3.91)^d^  45.45 (4.65)^f^  45.05 (3.44)^e^  36.0 (8.19)^d,f,e^ |
| ***H*** | 2.62 | 8.77 | 6.22 | 0.29 | 9.11 | 1.33 | 4.58 | 9.03 |
| ***p*** | 0.45 | 0.03* | 0.10 | 0.96 | 0.03* | 0.72 | 0.21 | 0.03* |
| ***Effect size*** | - | 2.750^d^/3.310^e^  2.427^f^ | - | - | 0.498^c^/1.081^d^  1.044^f^ | - | - | 1.572^d^/1.440^e^  1.419^f^ |
| **Housing**  Live alone (n=61)  Live with one other person (n=142)  Live with two other people (n=130)  Live with three or more people (n=126) | 7.77 (0.76)  7.75 (0.56)  7.86 (0.45)  7.83 (0.43) | 6.62 (1.14)^d^  6.49 (1.14)^c^  6.28 (1.37)^b^  5.78 (1.76)^b,d,c^ | 3.97 (2.03)  3.65 (2.05)  3.24 (1.86)  3.43 (1.84) | 7.11 (1.02)  7.15 (1.02)  7.02 (1.10)  7.06 (1.11) | 7.10 (1.00)  7.07 (0.89)  7.12 (0.77)  6.95 (0.88) | 7.03 (1.28)  7.06 (1.05)  7.17 (0.97)  6.98 (1.34) | 6.84 (1.41)  6.90 (1.34)  6.98 (1.36)  6.97 (1.33) | 46.44 (4.37)  46.08 (4.14)  45.67 (3.92)  44.99 (4.77) |
| ***H*** | 4.76 | 19.94 | 6.66 | 1.94 | 3.64 | 4.31 | 2.09 | 8.01 |
| ***p*** | 0.46 | <0.01* | 0.25 | 0.86 | 0.60 | 0.51 | 0.84 | 0.16 |
| ***Effect size*** | - | 0.317^b^/0.479^c^  0.567^d^ | - | - | - | - | - | - |

**M:** Mean; **SD:** Standard Deviation; **H**: Kruskall-Wallis test; **p:** Probability of significance; *****Statistically significant difference; **a**: Difference found between groups 1 and 2; **b:** Difference found between groups 2 and 3; **c:** Difference found between groups 1 and 3; **d**: Difference found between groups 1 and 4; **e:** Difference found between groups 3 and 4; **f:** Difference found between groups 2 and 4. The effect size was calculated using *Cohen's D* for parametric data. For nonparametric data, the Z value generated by the *Mann Whitney* or *Kruskall Whallis* test was divided by the value of the sample analyzed.

**Table 5**

*Analysis of the accuracy of each item in the set of facial expressions*

| **Item** | **If item is deleted** | | **Item-rest correlation** | ***M*** | ***DP*** |
| --- | --- | --- | --- | --- | --- |
|  | ***McDonald's ω*** | ***Cronbach's α*** |  |  |  |
| 1 Happiness | 0.681 | 0.697 | 0.156 | 0.924 | 0.266 |
| 2 Neutral | 0.681 | 0.698 | 0.144 | 0.924 | 0.266 |
| 3 Sadness | 0.678 | 0.697 | 0.164 | 0.832 | 0.374 |
| 4 Fear | 0.690 | 0.705 | 0.040 | 0.248 | 0.433 |
| 5 Happiness | 0.683 | 0.701 | 0.026 | 0.993 | 0.081 |
| 6 Anger | 0.682 | 0.700 | 0.079 | 0.974 | 0.160 |
| 7 Disgust | 0.681 | 0.700 | 0.098 | 0.991 | 0.093 |
| 8 Neutral | 0.684 | 0.697 | 0.179 | 0.935 | 0.247 |
| 9 Happiness | 0.687 | 0.698 | 0.199 | 0.987 | 0.114 |
| 10 Surprise | 0.666 | 0.689 | 0.283 | 0.667 | 0.472 |
| 11 Neutral | 0.688 | 0.708 | 0.030 | 0.634 | 0.482 |
| 12 Disgust | 0.690 | 0.699 | 0.157 | 0.996 | 0.066 |
| 13 Surprise | 0.682 | 0.700 | 0.073 | 0.987 | 0.114 |
| 14 Neutral | 0.685 | 0.698 | 0.151 | 0.867 | 0.340 |
| 15 Disgust | 0.692 | 0.705 | 0.065 | 0.649 | 0.478 |
| 16 Fear | 0.683 | 0.699 | 0.142 | 0.296 | 0.457 |
| 17 Sadness | 0.688 | 0.704 | 0.082 | 0.420 | 0.494 |
| 18 Happiness | 0.682 | 0.700 | 0.075 | 0.985 | 0.123 |
| 19 Disgust | 0.676 | 0.695 | 0.214 | 0.876 | 0.330 |
| 20 Surprise | 0.679 | 0.698 | 0.148 | 0.935 | 0.247 |
| 21 Disgust | 0.677 | 0.695 | 0.197 | 0.804 | 0.397 |
| 22 Neutral | 0.680 | 0.695 | 0.204 | 0.765 | 0.425 |
| 23 Sadness | 0.663 | 0.688 | 0.314 | 0.815 | 0.389 |
| 24 Anger | 0.678 | 0.697 | 0.238 | 0.980 | 0.139 |
| 25 Fear | 0.662 | 0.688 | 0.304 | 0.349 | 0.477 |
| 26 Anger | 0.679 | 0.697 | 0.158 | 0.928 | 0.259 |
| 27 Happiness | 0.679 | 0.694 | 0.262 | 0.956 | 0.204 |
| 28 Anger | 0.689 | 0.708 | 0.030 | 0.362 | 0.481 |
| 29 Disgust | 0.681 | 0.699 | 0.157 | 0.996 | 0.066 |
| 30 Sadness | 0.670 | 0.692 | 0.249 | 0.721 | 0.449 |
| 31 Neutral | 0.685 | 0.696 | 0.209 | 0.941 | 0.236 |
| 32 Anger | 0.682 | 0.693 | 0.330 | 0.967 | 0.178 |
| 33 Fear | 0.654 | 0.683 | 0.362 | 0.473 | 0.500 |
| 34 Surprise | 0.680 | 0.697 | 0.158 | 0.946 | 0.227 |
| 35 Fear | 0.665 | 0.690 | 0.279 | 0.468 | 0.500 |
| 36 Fear | 0.654 | 0.683 | 0.373 | 0.706 | 0.456 |
| 37 Surprise | 0.676 | 0.696 | 0.191 | 0.739 | 0.440 |
| 38 Happiness | 0.683 | 0.702 | -0.033 | 0.991 | 0.093 |
| 39 Anger | 0.681 | 0.699 | 0.125 | 0.915 | 0.279 |
| 40 Sadness | 0.674 | 0.694 | 0.212 | 0.813 | 0.391 |
| 41 Surprise | 0.677 | 0.696 | 0.177 | 0.885 | 0.320 |
| 42 Neutral | 0.682 | 0.697 | 0.167 | 0.895 | 0.306 |
| 43 Sadness | 0.678 | 0.698 | 0.164 | 0.980 | 0.139 |
| 44 Fear | 0.683 | 0.701 | 0.089 | 0.176 | 0.382 |
| 45 Disgust | 0.684 | 0.699 | 0.135 | 0.797 | 0.402 |
| 46 Fear | 0.663 | 0.689 | 0.304 | 0.800 | 0.401 |
| 47 Surprise | 0.670 | 0.692 | 0.299 | 0.919 | 0.273 |
| 48 Sadness | 0.666 | 0.690 | 0.313 | 0.887 | 0.317 |
| 49 Sadness | 0.666 | 0.690 | 0.279 | 0.786 | 0.410 |
| 50 Surprise | 0.682 | 0.699 | 0.170 | 0.989 | 0.104 |
| 51 Disgust | 0.683 | 0.700 | 0.079 | 0.972 | 0.166 |
| 52 Anger | 0.680 | 0.699 | 0.131 | 0.983 | 0.131 |
| 53 Happiness | 0.686 | 0.699 | 0.127 | 0.987 | 0.114 |
| 54 Anger | 0.679 | 0.697 | 0.176 | 0.946 | 0.227 |
| 55 Neutral | 0.687 | 0.695 | 0.301 | 0.974 | 0.160 |
| 56 Happiness | 0.682 | 0.701 | 0.038 | 0.983 | 0.131 |

**M:** Mean; **SD:** Standard Deviation

Final stimuli of the constructed face database

* For more details and access to the high-resolution database for research use, please contact the corresponding author


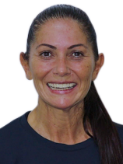

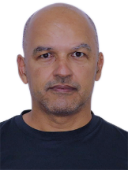

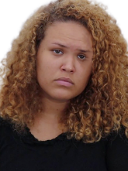

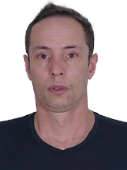

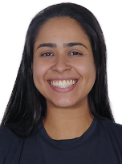

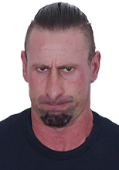

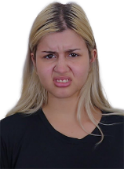

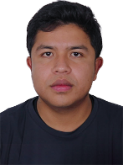

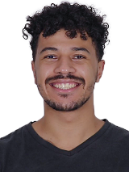

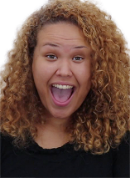

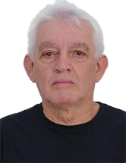

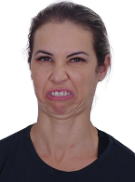

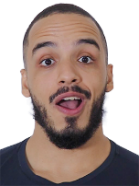

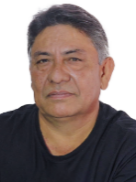

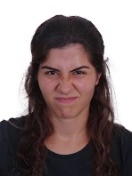

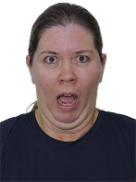

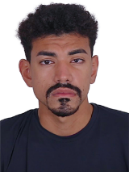

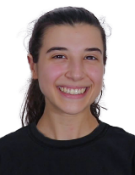


1 2 3 4 5 6 7 8 9 10 11 12 13 14 15 16 17 18


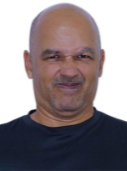

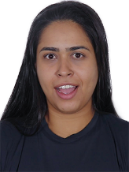

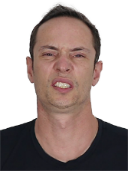

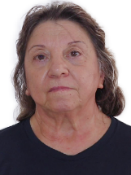

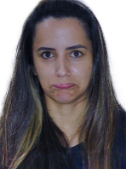

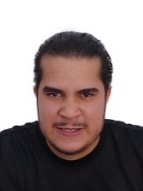

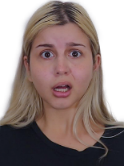

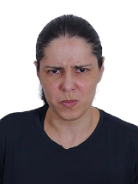

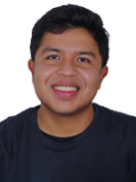

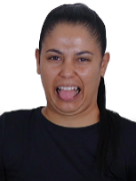

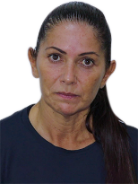

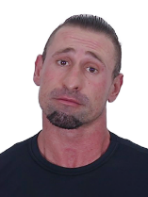

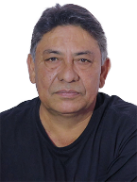

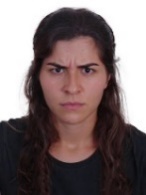

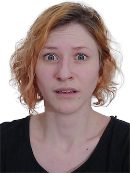

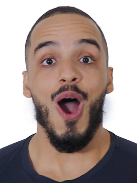

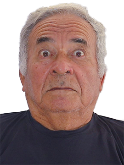

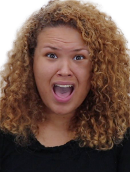


19 20 21 22 23 24 25 26 27 28 29 30 31 32 33 34 35 36


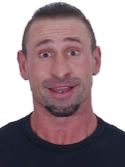

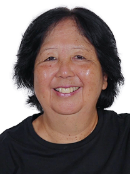

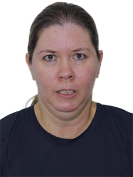

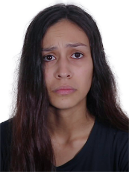

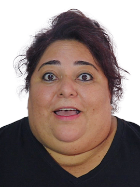

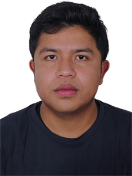

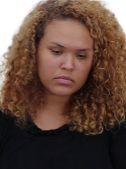

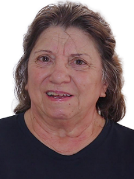

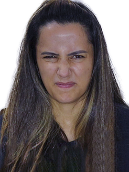

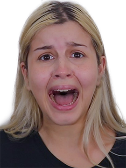


37 38 39 40 41 42 43 44 45 46


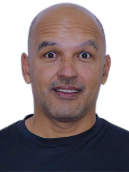

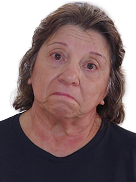

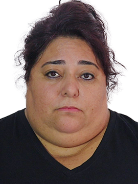

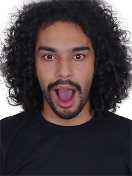

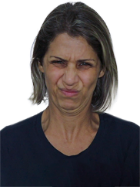

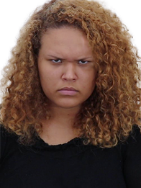

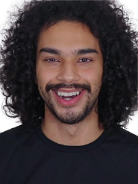

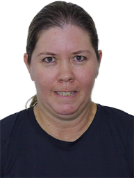

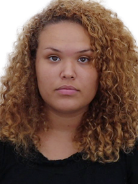

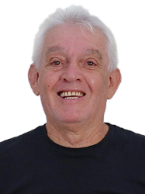


47 48 49 50 51 52 53 54 55 56
